# Prognostic Performance of Kidney Volume Measurement for Polycystic Kidney Disease: A Comparative Study of Ellipsoid vs. Manual Segmentation

**DOI:** 10.1038/s41598-019-47206-4

**Published:** 2019-07-29

**Authors:** Beili Shi, Pedram Akbari, Marina Pourafkari, Ioan-Andrei Iliuta, Elsa Guiard, Crystal F. Quist, Xuewen Song, David Hillier, Korosh Khalili, York Pei

**Affiliations:** 1 Division of Nephrology and University Health Network and University of Toronto, Toronto, Ontario Canada; 2 Department of Medical Imaging, University Health Network and University of Toronto, Toronto, Ontario Canada; 3 Canadian Institutes of Health Research (CIHR) Strategy for Patient Oriented Research Program (SPOR) affiliated with the Division of Nephrology, University Health Network and University of Toronto, Toronto, Ontario, Canada

**Keywords:** Kidney, Polycystic kidney disease

## Abstract

Total kidney volume (TKV) is a validated prognostic biomarker for risk assessment in autosomal dominant polycystic kidney disease (ADPKD). TKV by manual segmentation (MS) is the “gold standard” but is time-consuming and requires expertise. The purpose of this study was to compare TKV-based prognostic performance by ellipsoid (EL) vs. MS in a large cohort of patients. Cross-sectional study of 308 patients seen at a tertiary referral center; all had a standardized MRI with typical imaging of ADPKD. An experienced radiologist blinded to patient clinical results performed all TKV measurements by EL and MS. We assessed the agreement of TKV measurements by intraclass correlation(ICC) and Bland-Altman plot and also how the disagreement of the two methods impact the prognostic performance of the Mayo Clinic Imaging Classification (MCIC). We found a high ICC of TKV measurements (0.991, p < 0.001) between EL vs. MS; however, 5.5% of the cases displayed disagreement of TKV measurements >20%. We also found a high degree of agreement of the individual MCIC risk classes (i.e. 1A to 1E) with a Cohen’s weighted-kappa of 0.89; but 42 cases (13.6%) were misclassified by EL with no misclassification spanning more than one risk class. The sensitivity and specificity of EL in distinguishing low-risk (1A-B) from high-risk (1C-E) MCIC prognostic grouping were 96.6% and 96.1%, respectively. Overall, we found an excellent agreement of TKV-based risk assessment between EL and MS. However, caution is warranted for patients with MCIC 1B and 1C, as misclassification can have therapeutic consequence.

## Introduction

Autosomal dominant polycystic kidney disease (ADPKD) is the most common monogenic kidney disorder worldwide with a life-time risk of approximately 1:1,000 and the fourth leading cause of end stage renal disease (ESRD) in North America^1,2^. Mutations of two genes, *PKD1* and *PKD2*, account for the majority of genetically resolved cases in ADPKD. Recent studies have delineated a strong genotype-phenotype correlation with the most severe kidney disease associated with *PKD1* protein-truncating mutations, intermediate disease severity associated with *PKD1* non-truncating mutations, and mild disease associated with *PKD2* mutations^3–5^. Additionally, patients with complex biallelic or digenic mutations have more severe disease while those with no detectable mutations, mild disease^4–6^. At the clinical level, replacement of kidney parenchyma by increasing number and size of cysts with age is a typical feature of ADPKD, eventually leading to ESRD in a majority of patients. Kidney function measurements, such as serum creatinine, estimated glomerular filtration rate (eGFR) and creatinine clearance are not sensitive biomarkers for assessing disease severity or progression in ADPKD, as these measurements typically remain within or close to normal range until late in the clinical course^7^.

The Consortium for Radiologic Imaging Studies of Polycystic Kidney Disease (CRISP) have shown that total kidney volume (TKV) in ADPKD expands quasi-exponentially during adult life at an average rate of 5% per year albeit with considerable variability between patients and is a sensitive biomarker predicting kidney disease progression^8,9^. Both the Food and Drug Administration (FDA) and European Medicines Agency (EMA) have recently accepted TKV as a prognostic biomarker for enrichment of patients at high-risk for progression for clinical trials^10^. Using age- and height-adjusted TKV, the Mayo Clinic imaging classification (MCIC) provides a validated TKV-based risk assessment tool for selecting “high-risk” patients, defined as class 1C-1E, for clinical trials^11,12^. Moreover, with the approval of Tolvaptan as the first disease modifier drug for treatment of “high-risk” patients with ADPKD in multiple countries, TKV-based risk assessment assumes an increasingly important role for patient management in the clinical setting^13–15^.

Currently, TKV measurement with magnetic resonance (MR) or computed tomography (CT) images by manual segmentation (MS) is considered the “gold standard”. However, this method dictates manual tracing of each kidney outline slice by slice and thus, is labor-intensive and requires radiological expertise^16,17^. By contrast, TKV derived using an ellipsoid (EL) formula requires only measurement of the three orthogonal axes of each kidney and is much simpler but less accurate^11^. To encourage a more practical translation to the clinical setting, the MCIC uses EL-derived TKV for its risk assessment; however, this approach has not been well validated externally. In this study, we compared the agreement of TKV measurements by EL vs. MS in a large cohort of patients with ADPKD and evaluated how the disagreement of TKV measurements by the two methods impact on the prognostic performance of MCIC.

## Materials and Methods

### Study patient selection

The study cohort comprised 357 consecutive patients seen at a regional PKD center between April 1, 2011 and March 31, 2017; they were referred by more than 100 academic and community nephrologists from a metropolitan area with population of approximately 6.4 million. All except 3% of this cohort participated in the current study and provided informed consent according to a pre-specified research protocol approved by the institutional review board at the University Health Network in Toronto. All study patients were aged 18 years or older, diagnosed with ADPKD by renal imaging^18,19^ and/or genetic testing^4^, and underwent renal imaging by MRI or CT. Patients with atypical (i.e. MCIC Class 2) or non-ADPKD cystic kidney disease^11,20^ were excluded.

### Study protocol

Demographic and clinical information including blood pressure, serum creatinine, estimated GFR by the CKD-EPI equation, mutation class, and height-adjusted TKV were collected from all study patients.

All the research methods (i.e. genetic testing and imaging studies) used in this study were performed in accordance with guidelines and regulations at the Toronto General Hospital.

All but 5 study patients underwent a standardized protocol using a 1.5-T MRI scanner (GE Healthcare, Milwaukee, WI). Coronal T2–weighted single–shot fast spin echo MR images with fat saturation were used for TKV calculation with the following parameters: 0.59- to 1.41-mm/pixel resolution in plane, 3-mm slice thickness, 90° flip angle, and 500–1491 ms/82–101 ms repetition time/echo time. Five patients with contraindication to MRI (i.e. claustrophobia or metal parts in body) had contrast enhanced CT (Canon (formerly Toshiba) Medical Systems Corporation, Otawara, Japan) imaging instead (64 detector row, reconstructed coronally using 3 mm slice thickness). All DICOM files from MRI and CT were downloaded into a workstation and inspected to confirm complete coverage of both kidneys and image quality. One experienced radiologist (M.P.) with over 10 years of experience, blinded to the patient clinical and genetic results performed all TKV measurements by MS and EL at two separate sittings. From each set of abdominal MR images, the boundary of each kidney was manually delineated slice by slice using a commercially available software (Vitrea, Vital Images, version 6.9.1). Kidney volume was calculated by summing the products of the renal area and slice thickness. Non-renal parenchymal tissue (e.g. renal hilum) were excluded from the measurements. TKV measurements by EL was performed as follows: for each kidney, length was measured as the average maximal longitudinal diameter measured in the coronal and sagittal planes, width, from the transversal image at maximum transverse diameter, and depth, from the same image perpendicular to the width measurement, all in millimeters. TKV was calculated by using the sum of the left and right kidney volumes, derived by the equation: π/6 * (coronal length + sagittal length)/2 * width * depth /1,000. Height-adjusted TKV and age were used to generate a MCIC risk class (i.e. 1A-1E) for all study patients^11^.

Genetic testing was performed in a single research laboratory in Toronto using a validated long range PCR protocol and bidirectional sequencing of coding region and splice junctions of *PKD1* and *PKD2*^4,21^. All nonsense, frameshift, and canonical splice site mutations were grouped as protein-truncating mutations, and nonsynonymous missense or atypical splice site mutations were grouped as non-truncating mutations^4^. All non-truncating mutations and inframe insertions/deletions were evaluated for their potential pathogenicity using prediction algorithms (Align GVGD, PolyPhen-2, SIFT, PROVEAN, and Human Splicing Finder), review of the PKD mutation database (http://pkdb.mayo.edu), and by segregation analysis with additional affected family members when possible^4^. We also screened all mutation-negative patients by multiplex ligation–dependent probe amplification to detect large gene rearrangements^21^.

### Statistical analysis

Cohen’s Kappa value, specificity, sensitivity, positive predictive value, false positive rate, false negative rate, and accuracy were all calculated in Microsoft Office Excel 2016 for Windows. All other analyses were performed using the statistical package for the social sciences (SPSS software (Version 22.0, SPSS Inc., Chicago, IL). Continuous variables are expressed as means ± standard deviation (SD), whereas non-normally distributed variables, as median with interquartile range (IQR). Categorical variables were expressed as percentages. Differences between MS and EL was tested using a 2-tail t-test for normally distributed and a chi-squared tests for categorical variables. Intra-class correlation coefficient of TKV measured by EL and MS was determined in the total cohort. The agreement of TKV measurements by EL and MS was assessed by Bland-Altman plots, with calculation of agreement limits (95% confidence interval). A 2-sided P < 0.05 was considered to indicate statistical significance.

## Results

### Study patient characteristics

**Figure 1:**
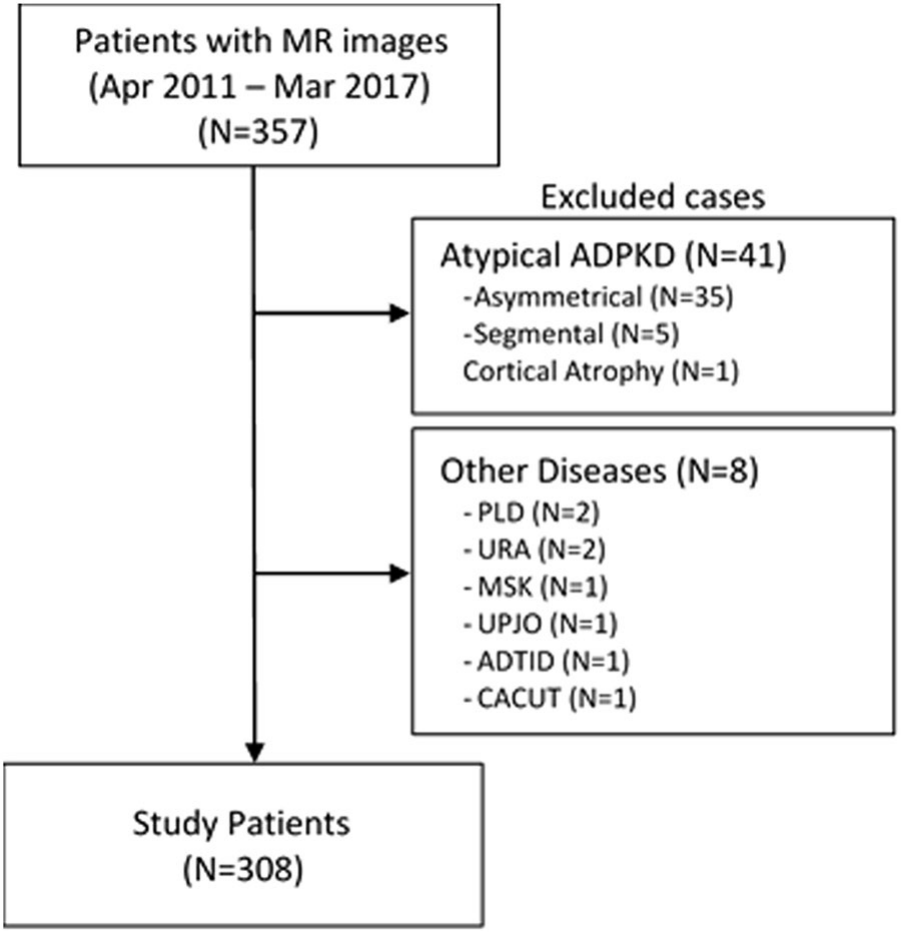
Assembly of study patient cohort. Patients with atypical renal imaging (i.e. Mayo Clinic Imaging Class 2) and non-ADPKD cystic kidney diseases were excluded from analysis. PLD: polycystic liver disease; URA: Unilateral renal agenesis; MSK: Medullary sponge kidney, UPJO: Ureteropelvic junction obstruction, ADTID: Autosomal dominant tubulointerstitial disease, CAKUT: Congenital Anomalies of the Kidney and the Urinary Tract.

**Figure 2:**
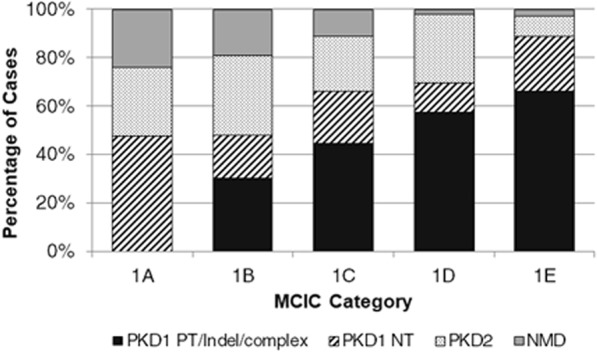
Enrichment of high-risk mutation classes in patients at high risk by Mayo Clinic Imaging Classification (MCIC). There is a significant association between high-risk mutation classes (i.e. *PKD1* PT/indel/complex mutations) and MCIC (i.e. 1 C, 1D, 1E) (*X*^2^ = 48.03, P < 0.001 by chi-square analysis).

The study cohort comprised 308 patients after exclusion of 41 cases with atypical renal imaging pattern (i.e. MCIC 2 A or 2B) and 8 cases of non-ADPKD renal cystic diseases (Fig. 1). The clinical characteristics of the study patients are shown in Table 1. Overall, their mean age was 44.9 years with 48% male and 54.9% hypertensive. Their median TKV as measured by MS was 1.25 L and their mean eGFR was 76.9 ml/min/1.73 m^2^. Overall, 69.5%, 21.4%, and 9.1% of our patients had chronic kidney disease (CKD) stages 1–2, 3, and 4–5, respectively; and approximately 1/3 of each had MCIC risk class 1A-1B, 1C, and 1D-1E, respectively. As expected, we found a positive correlation between TKV, age, blood pressure, serum creatinine, and a negative correlation between TKV and eGFR (Table 2). Patients with higher MCIC risk classes (1A-1B, to 1C, and 1D-1E) exhibited more advanced stages of CKD (Table 1). Similarly, patients with higher MCIC risk classes (1A-1B, to 1C, and 1D-1E) were also enriched with mutation classes (i.e. *PKD1* truncating mutations, *PKD1* inframe indels, and complex bilineal or digenic mutations) that are typically associated with more rapid rate of ADPKD progression (Fig. 2)^4,5,12^.

**Table 1:** Clinical characteristics of study cohort at the time of renal imaging.

	Total Patients (N = 308)	MCIC		
		1A-1B (N = 103)	1 C (N = 99)	1D-1E (N = 106)
Age at MRI	44.9 ± 13.9	47.8 ± 13.6	46.1 ± 14.6	39.4 ± 11.7
Gender (M:F)	1.00:1.10	1.00:1.28	1.00:1.75	1.65:1.00
Scr (µmol/L)	88 [72–118]	80 [66–93]	93 [71–117]	102 [77–143]
eGFR (mL/min/1.73 m^2^)	76.9 ± 30.4	84.9 ± 26.1	73.4 ± 31.8	72.8 ± 33.5
SBP (mmHg)	122 [118–130]	120 [114–130]	120 [120–130]	122 [119–134]
DBP (mmHg)	80 [75–84]	80 [70–80]	80 [78–85]	80 [77–85]
Hypertension (n, %)	155/282 (54.9)	43/89 (48.3)	52/94 (55.3)	60/99 (60.6)
TKV by EL (mL)	1275 [760–2119]	631 [483–995]	1316 [913–2089]	2494 [1429–3585]
TKV by MS (mL)	1250 [730–2122]	662 [494–958]	1352 [892–2003]	2457 [1477–3367]
CKD 1–2 (n, %)	214 (69.5)	84 (81.6)	64 (64.6)	66 (62.3)
CKD 3 (n, %)	66 (21.4)	13 (12.6)	26 (26.3)	27 (25.5)
CKD 4–5 (n, %)	28 (9.1)	6 (5.8)	9 (9.1)	13 (12.2)

Scr, serum creatinine; eGFR, estimated glomerular filtration rate; SBP, Systolic Blood Pressure; DBP, Diastolic Blood Pressure; TKV, Total Kidney Volume; EL, Ellipsoid formula; MS, Manual Segmentation; CDK, Chronic kidney disease. Age and eGFR are presented as mean ± standard deviation; Scr, BP, and TKV measurements are presented as median [IQR].

**Table 2:** Correlation between TKV by MS and clinical variables.

Parameters	TKV by MS (L)
	r
Age (y)	0.387
Systolic BP (mmHg)	0.317
Diastolic BP (mmHg)	0.290
Scr (μmol/L)	0.587
eGFR (ml/min/1.73 m^2^)	−0.604
TKV by EL (L)	0.990

TKV, Total Kidney Volume; EL, Ellipsoid formula; MS, Manual Segmentation.

### Comparing the agreement of TKV by EL vs. MS

**Figure 3:**
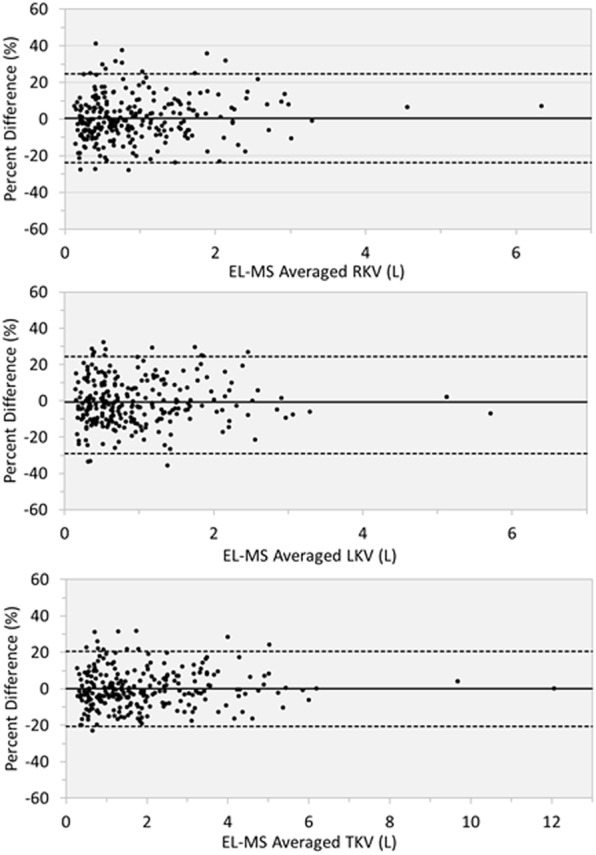
Bland-Altman plots comparing percent difference in kidney volumes between ellipsoid (EL) vs. manual segmentation (MS). Kidney volumes were calculated by averaging the EL and MS readings for each patient. Disagreement exceeding 20% were found in 11.4% (n = 35), 10.7% (n = 33) and 5.5% (n = 17) of the measurements for left (LKV), right (RKV), and total kidney volume (TKV), respectively.

We found a high intraclass correlation coefficient of TKV measurements (0.991; 95% confidence interval [95% CI], 0.989 to 0.993) between EL and MS. Figure 3 shows the results of the Bland–Altman plots of TKV between the EL and MS methods. We found a mean percent difference of −0.6% indicating minimal systematic bias of over- or under-estimation of TKV by EL. However, we found a disagreement of the volume measurements for the left, right, and both kidneys of greater than 20% in 11.4% (n = 35), 10.7% (n = 33) and 5.5% (n = 17) of cases, respectively. We also examined the effects of age on the agreement between the EL and MS methods by dividing our cohort into two strata: 18–30 vs. 31–79 years of age. These data are presented in Bland-Altman plots (Fig. S1**)** which show no significant percent differences in KV measurements by the two methods between the age strata (P = 0.15 by t-test).

### Comparing the prognostic performance of MCIC by EL vs. MS

**Figure 4:**
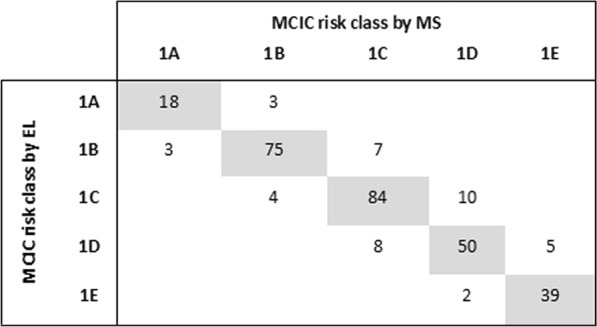
Comparison of MCIC risk class by ellipsoid (EL) versus manual segmentation (MS). In total, we found 42 (13.6%) patients were misclassified into either one higher or lower risk class by Mayo Clinic Imaging Classification (MCIC) when TKV was measured by EL instead of MS. Among these 42 cases, 15 cases (35.7% of all misclassified cases) belong to the risk class 1 C by MS.

To assess how TKV measurement errors by EL might impact the prognostic performance by MCIC, we tabulated the concordant and discordant cases across the five risk classes using the assignment by MS as the “gold standard” (Fig. 4 for overall cohort; Tables S1 and S2 for patients of 18–30 years and 31–79 years of age, respectively). We found that a total of 13.7% (42/308) of misclassified cases; 5.5% (17/308) were incorrectly assigned to a lower risk class and 8.1% (25/308), to a higher risk class. Investigating the performance of EL as a function of age revealed that misclassification rate decreases in older patients (i.e. 18% for 18–30, vs. 15% for 31–50 and 11% for 51–79 years) (Table 3, Tables S1 and S2). The positive predictive values (PPV) for correct assignment of the individual risk class by EL were 85.7% for 1A; 91.4% for 1B; 84.9% for 1C; 80.6% for 1D; and 88.6% for 1E. Overall, we found concordance of MCIC risk class in 266/308 of patients, which translates to an accuracy of 86.4% by EL. However, the accuracy of the younger patients (aged 18–30 years) was lower compared to the older patients (aged 31–79 years) at 82.1% vs. 87.4%, respectively (Table 4). None of the misclassified cases spanned more than one risk class and 35.7% (n = 15) of them belong to risk class 1C. Overall, these findings are consistent with a high weighted Cohen’s Kappa coefficient (0.89; 95% CI 0.86–0.92).

**Table 3:** MCIC misclassified patients by age strata.

	Age (Years)		
	18–30	31–50	51–79
N misclassified	10/57	21/151	11/100
Percentage (%)	18	15	11

**Table 4:** Performance of EL vs. MS in younger vs. older patients.

	Age Category (Years)	
	18–30 (N = 57)	31–79 (N = 251)
Sensitivity	0.894	0.987
Specificity	0.900	0.968
FPR	0.023	0.019
FNR	0.357	0.022
Accuracy	82.1%	87.4%

FPR: false positive rate; FNR: false negative rate.

For clinical prognostication using MCIC, it has been proposed that classes 1A-1B be used to identify low-risk while classes 1C-1E, for high-risk patients with rapid progression^15^. Using these two broad prognostic groupings (i.e. 1A-1B vs. 1C-E) as an outcome, we found a sensitivity and specificity for correct risk assignment by EL (in the overall cohort) to be 96.6%, of 96.1%, respectively. By contrast, both the sensitivity (0.894 vs. 0.987) and specificity (0.900 vs. 0.968) for correct risk assignment by EL were respectively reduced in the younger (aged 18–30 years) as compared to older (aged 31–79 years) patients (Table 4). Similarly, both the false positive and negative rates were higher in the younger patients.

## Discussion

Recent approval of Tolvaptan as the first disease modifier drug for treatment of ADPKD in multiple countries heralds a new era when risk assessment will become a critical and integral component of clinical management for this disease^13–15^. TKV-based risk assessment as employed by the MCIC provides a robust and validated clinical approach to identify patients at “high-risk” for progression to ESRD who are most likely to benefit from disease-modifier drug treatment; at the same time, “low-risk” patients thus identified can be reassured and managed with conservative measures^11,12^. Our findings of enriched “high-risk” characteristics (i.e. positive association with increasing age, blood pressure, serum creatinine, and high-risk mutation classes, and negative association with eGFR, Table 2) in patients with large cystic kidneys are corroborative of previous studies supporting the prognostic value of TKV-based assessment^8–12^. In this context, the MCIC further allows identification of “high-risk” patients who have not yet developed advanced CKD stages due to their younger age, but who may benefit the most from disease-modifier drug treatment^15^.

TKV measurement by MS in ADPKD is currently time-consuming, requires radiological expertise including the use of specialized computer software, and remains challenging outside specialized centers. While this is essential in clinical trials to provide accurate assessment of the therapeutic effects, exact TKV measurement may not be necessary for risk assessment. In this regard, the use of EL-derived TKV by the MCIC significantly reduces the technical complexity and time required, and allows for wider clinical translation^11^. However, EL-derived TKV measurements are less accurate^22,23^. In this study, we examined the performance of EL based TKV by analyzing their predictive MCIC risk assessments in 308 patients over a wide range of disease severity. Compared to MS, we found that EL-derived TKV generally performed well with low bias, high precision and accuracy. Moreover, ICC of the TKV measurements and PPVs for correct assignment of individual MCIC risk classes by EL were all very high. Nevertheless, a disagreement of TKV measurement exceeding 20% was found in 5.5% of cases. Clinically, two prognostic groupings (i.e. 1A-1B vs. 1C-1E) based on individual MCIC classes have been proposed to separate “low-risk” from “high-risk” patients^15^. Misclassification of class 1B or 1C by EL potentially may have the greatest negative impact on clinical management including the use of a disease-modifier drug. Caution may be warranted for patients with these two risk classes; additional risk factors such as ADPKD mutation class^3–5^, family history of PKD disease severity^24^, rate of loss of eGFR^25^, or repeat TKV measurement after 12 months may help to improve the confidence of assessment for patients in class 1C, while repeat TKV assessment after 12 months may identify high-risk patients misclassified as class 1B. The issue of MCIC misclassification is further exacerbated by a narrower TKV range that defines each MCIC class in the younger patients^11^. Thus, measurement errors in TKVs by EL had a greater negative impact on the accuracy of MCIC risk classification in the younger (aged 18–30 years) compared to older (aged 31–79 years) patients.

The strengths of our study include a large cohort of well-characterized patients who underwent a standardized MRI protocol and their TKV measured using MS and EL by a single experienced radiologist. However, this study has several limitations. First, since our study involved a single center with only one radiology reader, we are unable to evaluate the impact of TKV measurement by different readers and multiple sites. We expect that there may be more disagreement in the TKV measurements under these latter scenarios which can be minimized by standardization of the MRI protocol and training of the readers. Second, we are unable to evaluate the validity of serial EL TKV measurements for risk assessment over time given that our study was cross-sectional. Lastly, the challenge of TKV measurements by MS may be overcome in the near future by recent innovations, such as semi- to full-automation by specialized computer software and the “deep learning” technologies^26,27^. Thus, TKV by MS may eventually become the standard option, where additional information from MR images such as texture analyses to delineate and quantify the functional kidney mass may also be added to improve risk assessment in ADPKD^28^. However, TKV via EL may still be attractive in centers that would not have the technology or expertise to use machine learning technologies.

In conclusion, TKV-based risk assessment is now an integral component of clinical management for ADPKD. We found that TKV by EL performed well with low bias, and high precision and accuracy when compared to MS. Although TKV measurement errors also resulted in the discordance of MCIC in 13.6% of our patients, their impact on MCIC is clinically most relevant in the misclassified patients with risk classes 1B and 1C. In the latter patients, incorporation of additional risk factors such as ADPKD mutation class, family history of PKD disease severity, rate of loss of eGFR, or repeat TKV measurement after 12 months may help to improve the confidence of their risk assessment. Moreover, younger patients aged 18–30 years are susceptible to a higher MCIC misclassification rate which may be minimized by using TKV derived from MS, rather than EL.

## Supplementary information

Supplementary Information

## Data Availability

The datasets generated during and/or analysed during the current study are available from the corresponding author on reasonable request.
