# Supplementary material for: Prognostic Performance of Kidney Volume Measurement for Polycystic Kidney Disease: A Comparative Study of Ellipsoid vs. Manual Segmentation
Source: Sci Rep. 2019 Jul 29;9:10996. doi: 10.1038/s41598-019-47206-4 (PMC6662759; doi:10.1038/s41598-019-47206-4)
Supplement: Supplementary file 1 — Supplementary Information [file 41598_2019_47206_MOESM1_ESM.pdf]

Supplementary Information:

Prognostic Performance of Kidney Volume Measurement for Polycystic Kidney Disease: A Comparative Study of Ellipsoid vs. Manual Segmentation.

Beili Shi,[1,2] Pedram Akbari,[1,2] Marina Pourafkari,[2,3] Ioan-Andrei Iliuta,[1] Elsa Guiard,[1] Crystal F. Quist,[1] Xuewen Song,[1] David Hillier,[4] Korosh Khalili,[2,5] York Pei.[1,5*]

[1]Division of Nephrology and [3]Department of Medical Imaging, University Health Network and University of Toronto, Toronto, Ontario, Canada.

[2]These authors contributed equally to this work; [4]patient partner from the CIHR SPOR CAN-SOLVE network (see acknowledgment); co-senior authors.[5]

Address correspondence to:
Korosh Khalili, MD
Department of Medical Imaging, Toronto General Hospital
585 University Ave., Toronto, ON M5G 2N2
Email: Korosh.Khalili@uhn.ca

or

York Pei, MD
Division of Nephrology, Toronto General Hospital
8N838, 585 University Avenue, Toronto, ON M5G 2N2
Email: york.pei@uhn.ca

Supplementary Information

Table S1. MCIC of patients aged 18-30 years classified by EL vs. MS

MCIC risk class by EL		MCIC risk class by MS				
		**1A**	**1B**	**1C**	**1D**	**1E**
	**1A**	2	1			
	**1B**		6	5		
	**1C**		1	14	1	
	**1D**			1	7	
	**1E**				1	18

Table S2. MCIC of patients aged 31-79 years classified by EL vs. MS

MCIC risk class by EL		**MCIC risk class by MS**				
		**1A**	**1B**	**1C**	**1D**	**1E**
	**1A**	16	2			
	**1B**	3	69	2		
	**1C**		3	70	9	
	**1D**			7	43	5
	**1E**				1	21

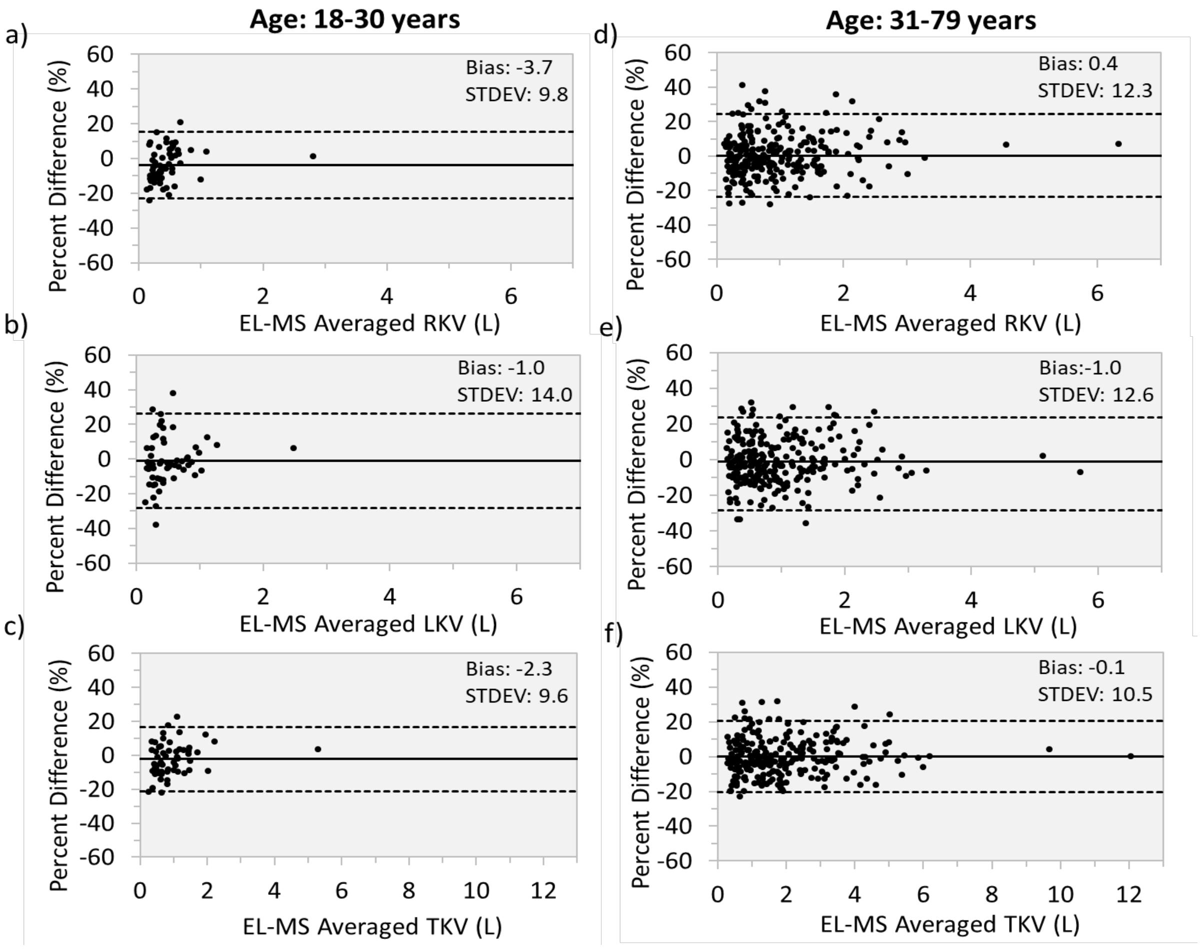

Figure S1. Bland-Altman plots of kidney volume (KV) measurements of patients aged 18-30 years (a-c) and 31-79 years (d-f). There were no significant percent differences of the KV measurements between the two age strata (P=0.15 by t-test).
